# Pulmonary Hypertension and Transbronchial Lung Biopsy: Does It Increase the Risk of Hemorrhage?

**DOI:** 10.7759/cureus.9084

**Published:** 2020-07-09

**Authors:** Bilal H Lashari, Megumi Asai, Waed Alswealmeen, Caitlin Hodge, Charis Ripley-Hager, Rajesh Kumar Patel

**Affiliations:** 1 Internal Medicine, Abington Hospital - Jefferson Health, Abington, USA; 2 Surgery, Abington Hospital - Jefferson Health, Abington, USA; 3 Pulmonary and Critical Care Medicine, Abington Hospital - Jefferson Health, Abington, USA

**Keywords:** transbronchial lung biopsy, pulmonary hypertension, complication, hemorrhage

## Abstract

Background

Bronchoscopy with transbronchial lung biopsy (TBLB) is commonly used as a diagnostic tool for pulmonary disease. Hemorrhage is a major complication of TBLB. While pulmonary hypertension (PH) is considered a risk factor, evidence supporting this is limited. In this study, we compare complications of TBLB in patients with PH to those without PH.

Material and methods

We performed a retrospective review of patients who underwent TBLB in our institution from January 2010 to May 2016. PH and non-PH groups were compared with respect to patient demographics, biopsy guidance, number of lobes biopsied (single or multiple), positive pressure ventilation, pre- and post-procedure diagnoses, and complications. Complications were defined as major hemorrhage, prolonged intubation, and reintubation within 72 hours from TBLB.

Results

The PH group had 45 patients with a mean age of 71 ± 14 years, and the non-PH group had 349 patients with a mean age of 63 ± 14 years. There were no significant differences with regards to gender, pre-procedure anticoagulation and antiplatelet agents, biopsy guidance, or number of lobes biopsied (p > 0.371). There was no significant difference in the occurrence of major hemorrhage between the two groups (p = 0.491). Prolonged intubation occurred more frequently in the PH group (p = 0.007).

Conclusions

There appears to be no increased risk of post-procedure hemorrhage with TBLB in patients with mild PH. There is, however, an increased risk of post-procedure prolonged intubation in these patients.

## Introduction

After Levin et al. described transbronchial lung biopsy (TBLB) using a fiber-optic bronchoscope in 1974, it has been done routinely in the field of pulmonary medicine to sample infiltrative parenchymal disease [1]. Although TBLB is usually a safe procedure, complications can occur during or after the biopsy. Major hemorrhage is one of the more feared complications related to TBLB, with a previously reported incidence rate of 0.2% to 25% [2-7]. Risk factors for TBLB include renal failure, anticoagulant and antiplatelet agent use, and lung transplant [3,8-11]. Although pulmonary hypertension (PH) is often considered a risk factor for hemorrhage, PH remains controversial as there are limited data available [8,12]. Wahidi et al. conducted a survey at the American College of Chest Physicians annual meeting in 2001 and found that 28.7% of physicians consider PH an absolute contraindication and 58.6% considered it a relative contraindication to TBLB [13].

In this study, we retrospectively reviewed 394 patients who underwent TBLB in our institution and compared postoperative complications between patients with and without PH.

## Materials and methods

Design

We retrospectively evaluated non-ventilator dependent patients who underwent TBLB between January 2010 and May 2016 in a single institution, and all patients were included in the analysis. The patients were classified into two groups based on a diagnosis of PH. The PH group included patients who were diagnosed with PH with an echocardiogram or right heart catheterization prior to, or within one year after the procedure. PH was defined as mean pulmonary artery pressure (mPAP) equal to or more than 25 mmHg on invasive catheterization, and right ventricular systolic pressure (RVSP) equal to or more than 40 mmHg on echocardiogram. Due to a paucity of data, further classification of PH into pulmonary arterial hypertension or PH due to cardiac or lung disease was not made. Some patients were electively intubated and were on positive pressure ventilation during the procedure at the discretion of the bronchoscopist and the anesthesiologist. Midazolam or propofol and fentanyl were used for sedation in all patients. Most patients who were intubated received rocuronium or succinylcholine during the induction phase. Topical lidocaine was used for patients with spontaneous breathing who were not intubated for the procedure. TBLB was performed under routine fluoroscopic guidance or electromagnetic navigational guidance using biopsy forceps. A routine chest x-ray was taken after the procedure in both groups.

We reviewed and compared patient demographics, antiplatelet agent or anticoagulant use before the procedure, renal insufficiency (blood urea nitrogen ≥ 30 mg/dL and/or creatinine ≥ 2 mg/dL), baseline pulmonary condition including chronic obstructive pulmonary disease (COPD) and pre-procedure oxygen requirement, type of ventilation during the procedure, number of lobes biopsied (single or multiple), pre- and post-procedure diagnoses, and post-procedure complications for the two groups. Post-procedure complications were defined as major hemorrhage, prolonged intubation (more than 72 hours post-procedure), and reintubation within 72 hours from TBLB. Major hemorrhage was defined as hemorrhage of more than 50 cc or hemorrhage requiring intubation for patients not already intubated, prolonged intubation, or reintubation. 

This study was exempt from Institutional Review Board approval under category 45 CFR 46.101 (b)(4).

Equipment

The fiberoptic bronchoscopy was performed using Olympus (Tokyo, Japan) BF-1T180, BF-XT160, BF-H190, and BF-Q190 scopes, with a 2.0-mm working channel for routine fluoroscopic-guided TBLB. An Olympus BF-1TH190 scope with a 2.8-mm working channel was used for electromagnetic navigational-guided TBLB. We used 1.8-mm alligator cup forceps for routine fluoroscopic biopsy and 1.7-mm cup forceps for electromagnetic navigational biopsy.

Statistical analysis

Chi-square and t-tests were used for statistical analysis. All tests were two-tailed. The tolerance for a type I error was 0.05. Missing data were excluded from the calculation of proportions unless otherwise specified. Data analysis was performed using IBM SPSS Statistics for Windows, Version 23.0 (IBM Corp., Armonk, NY).

## Results

A total of 394 patients were identified during the given search period. These patients were divided into PH and non-PH groups. The non-PH group had 348 patients with a mean age of 63 ± 14 years, 58.3% of whom were female. The PH group had 46 patients with a mean age of 71 ± 15 years, and 63.0% were female. The mean age was significantly higher in the PH group (p = 0.001). In the PH group, 36 patients were diagnosed with echocardiogram, whereas 10 patients had a diagnostic right-sided cardiac catheterization. Among those, 35 patients had mild, eight had moderate, and three had severe PH (Table 1). Mean mPAP was 33.2 ± 7.2 mmHg on catheterization, and mean RVSP was 47.6 ± 13.8 mmHg on echocardiogram. 

**Table 1:** Diagnostic modality and severity of pulmonary hypertension RSVP, right ventricular systolic pressure; RHC, right heart catheterization; mPAP, mean pulmonary artery pressure; echo, echocardiography.

	Patient, n
RVSP on echo, mmHg	36
Mild (40-50 mmHg)	27
Moderate (51-60 mmHg)	6
Severe (>60 mmHg)	3
mPAP on RHC, mmHg	10
Mild (25-40 mmHg)	8
Moderate (41-55 mmHg)	2
Severe (>55 mmHg)	0

Approximately two-thirds of the procedures were performed in an outpatient setting. Among those who had the procedure as an inpatient, four patients in the non-PH group and two in the PH group were admitted to the intensive care unit before the procedure. A total of 46 patients in the non-PH group and 13 patients in the PH group were on supplemental oxygen of two to six liters before the procedure; this was statistically significant (13.2% vs. 28.2% p = 0.014). Renal insufficiency was more common in the PH group (5.2% vs. 17.4%, p = 0.006). In patients with renal insufficiency, desmopressin was not given before the procedure, and the patients with end-stage renal disease on hemodialysis received hemodialysis as scheduled. More than 30% of patients in each group were on an antiplatelet agent and/or anticoagulation before the procedure. The most common antiplatelet agents were aspirin and clopidogrel. Clopidogrel was held for at least five days before the procedure. Aspirin was continued at the discretion of the physician who performed the biopsy. The most common anticoagulant was warfarin followed by heparin infusion and enoxaparin. All anticoagulation was held before the procedure, or the effect was reversed with reversal agents such as fresh frozen plasma and vitamin K. The choice to intubate the patient for the procedure was based on physician and patient preference regardless of the patient’s baseline respiratory condition. Ventilator settings were adjusted continuously by an anesthesiologist during the procedure. All ventilated patients were on positive end-expiratory pressure of at least 5 cmH_2_O during the procedure. The fraction of inspiratory oxygen was kept as high as 50% to 100% regardless of respiratory condition to ensure adequate oxygenation during the procedure. There were no differences in the rate of antiplatelet or anticoagulant use, platelet counts, ventilation during the procedure, number of lobes biopsied, or biopsy guidance in the two groups (Table 2).

**Table 2:** Demographics and details of procedures in 394 patients undergoing transbronchial lung biopsy COPD, chronic obstructive pulmonary disease; PH, pulmonary hypertension.

	Non-PH group (n = 348)	PH group (n = 46)	P-value	Total (n = 394)
Age, mean (years)	63.2	71.2	0.001	64.1
Female, n (%)	203 (58.3)	29 (63.0)	0.633	232 (58.9)
Male, n (%)	145 (42.7)	17 (37.0)		162 (41.1)
Inpatient, n (%)	107 (30.7)	21 (45.7)	0.460	128 (32.5)
COPD, n (%)	108 (31.0)	17 (37.0)	0.405	125 (31.7)
Supplemental O_2_, n (%)	46 (13.2)	13 (28.2)	0.014	59 (15.0)
Renal insufficiency, n (%)	18 (5.2)	8 (17.4)	0.006	26 (6.6)
Dialysis, n (%)	1 (0.3)	2 (4.3)	0.037	3 (0.8)
Antiplatelet use, n (%)	101 (29.0)	18 (39.1)	0.173	119 (30.2)
Cont. aspirin, n (%)	79 (22.7)	13 (28.2)	0.763	92 (23.4)
Anticoagulant use, n (%)	30 (8.6)	5 (10.1)	0.583	35 (8.9)
Platelet count, mean (x10^3^/µl)	275.61	276.29	0.968	275.7
Positive pressure ventilation, n (%)	308 (88.5)	43 (93.5)	0.450	351 (89.1)
Number of lobes biopsied				
Single lobe, n (%)	255 (73.3)	36 (78.3)	0.593	291 (73.9)
Multiple lobe, n (%)	93 (26.7)	10 (21.7)		103 (26.1)
Biopsy guidance				
Routine fluoroscopy, n (%)	230 (66.1)	32 (69.6)	0.740	262 (66.5)
Electronavigational, n (%)	118 (33.9)	14 (30.4)		132 (33.5)

TBLB was performed by a total of 11 pulmonologists, with two performing most of the procedures (Table 3). Pre-procedure diagnosis and post-procedure pathologic findings are summarized in Table 4. The most common indication for TBLB was an assessment of a lung mass or nodules in both groups (49.7% and 45.7%) followed by pulmonary infiltrates (21.8% and 37.0%). The most common post-procedure tissue diagnosis was cancer (15.1% and 20.9%) in both groups.

**Table 3:** Physicians who performed transbronchial lung biopsy PH, pulmonary hypertension.

	Non-PH group (n = 348)	PH group (n = 46)	Total (n = 394)
Physician A	237	28	265
Physician B	72	13	85
Physician C	5	2	7
Physician D	5	2	7
Physician E	5	1	6
Physician F	6	0	6
Physician G	6	0	6
Others	12	0	12

**Table 4:** Pre-procedure and post-procedure diagnoses BOOP, bronchiolitis obliterans organizing pneumonia; PH, pulmonary hypertension.

	Non-PH group (n = 348)	PH group (n = 46)	Total (n = 394)
Pre-procedure diagnosis			
Mass/nodules, n (%)	173 (49.7)	21 (45.7)	194 (49.2)
Pulmonary infiltration, n (%)	76 (21.8)	17 (37.0)	93 (23.6)
Suspected sarcoid, n (%)	28 (8.0)	3 (7.0)	31 (7.9)
Miscellaneous, n (%)	71 (20.4)	5 (10.9)	76 (19.3)
Post-procedure diagnosis			
Cancer, n (%)	53 (15.1)	9 (20.9)	62 (15.7)
Fibrosis, n (%)	10 (2.9)	1 (2.1)	11 (2.8)
Acute/chronic inflammation, n (%)	48 (13.8)	6 (13.0)	54 (14)
Granulomatous disease, n (%)	32 (9.2)	3 (6.5)	35 (8.9)
BOOP, n (%)	15 (4.3)	1 (2.1)	16 (4.1)
Miscellaneous, n (%)	26 (7.4)	1 (2.1)	27 (6.9)
Normal/non-diagnostic, n (%)	164 (47.1)	22 (47.8)	186 (47.2)

Complications are shown in Table 5. Severe hemorrhage occurred in seven patients in the non-PH group, with one intubated during the procedure to protect the airway. One patient in the PH group had severe bleeding and required prolonged intubation due to hemorrhage. The incidence of major hemorrhage was not statistically different between the two groups.

**Table 5:** Complications for patients undergoing transbronchial lung biopsy PH, pulmonary hypertension.

	Non-PH group (n = 348)	PH group (n = 43)	P-value	Total (n = 394)
Patient with one or more complications, n (%)	32 (9.1)	6 (13.0)	0.423	38 (9.6)
Hemorrhage, n (%)	7 (2.0)	1 (2.2)	1.000	8 (2.0)
Prolonged intubation/reintubation, n (%)	7 (2.0)	5 (10.9)	0.007	12 (3.0)

Seven patients had prolonged intubation or reintubation in the non-PH group, one due to bleeding, two due to COPD exacerbation, one due to mental status change, and two due to desaturation of unknown origin. In the PH group, five patients required prolonged intubation or reintubation due to bleeding, pneumonia, and airway edema. One patient in each group was on six liters of oxygen via nasal cannula before the procedure and underwent the procedure with planned post-procedure intubation for 24 hours. The incidence of major hemorrhage and prolonged intubation or reintubation was higher in the PH group but failed to reach statistical significance.

## Discussion

Flexible bronchoscopy and TBLB is a safe procedure with a low risk of mortality [14]. Hemorrhage is a commonly feared complication after TBLB with a risk of 0.2% to 25%, depending on the definition of bleeding [2-7]. The risk factors associated with major hemorrhage have been reported as thrombocytopenia, coagulopathy, status post lung transplantation, immunosuppression, and renal insufficiency [3,8-11,15].

PH is considered a risk factor for hemorrhage [8,13]. Chronic venous hypertension in the pulmonary circulation may cause submucosal vessel dilation, and elevation of pulmonary capillary pressure is a theoretical risk for a significant hemorrhage after transbronchial biopsy [16,17]. Although there is limited information available regarding the relationship between PH and actual risk of hemorrhage, 58.6% of physicians consider PH as, at least, a relative contraindication to TBLB [13]. There is a paucity of literature reporting association between PH and post-TBLB hemorrhage. Schulman et al. observed 37 TBLBs on cardiac transplant recipients and reported an association with PH and moderate hemorrhage [18]. In this study, all patients had a diagnostic right-sided cardiac catheterization. The incidence of the moderate hemorrhage (25 to 100 cc) was 15% in patients with mPAP more than 16 mmHg, whereas no major hemorrhage occurred in patients with mPAP ≤ 16 mmHg. Morris et al. assessed the risk of bleeding in the setting of PH in a sheep model and concluded that there is no increased risk of hemorrhage [19]. This was followed by a prospective study in patients with interstitial lung disease [20]. In this study, pulmonary artery systolic pressure (PASP) was estimated using echocardiogram, and 22 of 50 patients had evidence of PH with mean PASP of 42 ± 12 mmHg. Only one case of moderate hemorrhage (26 to 50 cc) occurred in the PH group and mild hemorrhage (11 to 25 cc) in the non-PH group. Although this study excluded patients with symptoms and radiologic evidence of PH, and more patients seem to fall in the mild PH group, there was no significant difference in the two groups regarding the risk of hemorrhage. Diaz-Guzman et al. retrospectively reviewed 45 patients with PH and 45 patients without PH who underwent diagnostic bronchoscopy [17]. Among the 45 patients with PH, 13 patients underwent catheterization, and others only had echocardiogram with an estimated RVSP; 60% of patients had moderate to severe PH. A total of 32 TBLBs were performed on patients with PH with no increased risk of bleeding. Diaz-Fuentes et al. also reviewed 107 patients with PH who underwent bronchoscopy, including 91 patients who had TBLB [21]. An echocardiogram was used to estimate RVSP and none had cardiac catheterization. In the PH group, 66% had RVSP from 36 to 55 mmHg. There was no increased risk in both minor and major hemorrhage when compared to the control group. 

Our study supported the results of the latter three studies. Although each study used different cut-off and severity classifications for PH, it seems there is general agreement in the literature that, at least for patients with mild PH, the risk of hemorrhage is not increased when performing TBLB. The uniqueness of our study is that most of our patients were intubated and some patients had longer procedures due to the use of electromagnetic navigational bronchoscopy. Even in these patients, there appears to be no increased risk of major bleed or complications of prolonged intubation in patients with PH.

There are several limitations with our study. Foremost, the small sample size and the primarily mild disease in PH group make extrapolation of results to more severe PH and general population difficult. Additionally, TBLB is a varied procedure with different risk profiles when performed with a navigation or standard approach, and the lack of sufficient patients in each group makes analysis of either approach difficult. Another limitation is the measurement of pulmonary arterial pressure. Although echocardiogram seems to be a decent tool in the estimation of right heart pressure in some studies, the vast majority of current literature does not support the use of echocardiogram for the diagnosis of PH, most of our patients did not have a direct measurement of pulmonary arterial pressure via catheterization, and RVSP may be under or overestimated [22]. Based on our data, and limitations of the study, larger prospective multicenter study may be required to validate our results. 

## Conclusions

Based on the findings of our study, it appears that a TBLB can be safely performed in mild PH patients without increasing risk of hemorrhage.
